# Cost-effectiveness of population screening for aortic stenosis

**DOI:** 10.1093/ehjqcco/qcae043

**Published:** 2024-05-22

**Authors:** Pouya Motazedian, Graeme Prosperi-Porta, Benjamin Hibbert, Hawre Jalal, Marino Labinaz, Ian G Burwash, Omar Abdel-Razek, Pietro Di Santo, Trevor Simard, George Wells, Doug Coyle

**Affiliations:** University of Ottawa Heart Institute, University of Ottawa, Ottawa, Ontario, Canada; School of Epidemiology and Public Health, University of Ottawa, Ottawa, Ontario, Canada; University of Ottawa Heart Institute, University of Ottawa, Ottawa, Ontario, Canada; Division of Cardiology, Mayo Clinic, Rochester, MN, USA; School of Epidemiology and Public Health, University of Ottawa, Ottawa, Ontario, Canada; University of Ottawa Heart Institute, University of Ottawa, Ottawa, Ontario, Canada; University of Ottawa Heart Institute, University of Ottawa, Ottawa, Ontario, Canada; University of Ottawa Heart Institute, University of Ottawa, Ottawa, Ontario, Canada; Division of Cardiology, Beth Israel Deaconess Medical Centre, Harvard University, Boston, MA, USA; University of Ottawa Heart Institute, University of Ottawa, Ottawa, Ontario, Canada; School of Epidemiology and Public Health, University of Ottawa, Ottawa, Ontario, Canada; Department of Medicine, The Ottawa Hospital, Ottawa, Ontario, Canada; Division of Cardiology, Mayo Clinic, Rochester, MN, USA; University of Ottawa Heart Institute, University of Ottawa, Ottawa, Ontario, Canada; School of Epidemiology and Public Health, University of Ottawa, Ottawa, Ontario, Canada; School of Epidemiology and Public Health, University of Ottawa, Ottawa, Ontario, Canada

**Keywords:** Aortic stenosis, Screening, Cost-effectiveness

## Abstract

**Aims:**

Aortic stenosis (AS) is a progressive disease predominantly affecting elderly patients that carries significant morbidity and mortality without aortic valve replacement, the only proven treatment. Our objective was to determine the cost-effectiveness of AS screening using transthoracic echocardiography (TTE) in a geriatric population from the perspective of the publicly funded healthcare system in Canada.

**Methods and results:**

Markov models estimating the cost-effectiveness ratio (ICER) for AS screening with a one-time TTE were developed. The model included diagnosed and undiagnosed AS health states, hospitalizations, transcatheter aortic valve replacement (TAVR), and post-TAVR health states. Primary analysis included screening at 70 and 80 years of age with intervention at symptom onset, with scenario analysis included for early intervention at the time of severe asymptomatic AS diagnosis. Monte Carlo simulation of 5000 replications was completed with a lifetime horizon and a 1.5% discount for costs and outcomes.Screening for AS at the age of 70 years was associated with an ICER of $156 722, and screening at 80 years of age was associated with an ICER of $28 005, suggesting that screening at 80 years of age is cost-effective when willingness-to-pay per QALY is $50 000. Scenario analysis with early intervention was not cost-effective, with an ICER of $142 157 at 70 years and $124 651 at 80 years.

**Conclusion:**

Screening for AS at 80 years of age with a one-time TTE, in a Canadian population, improves quality of life and is cost-effective in a publicly funded healthcare system providing, TAVR is reserved for symptomatic patients.

## Graphical Abstract

**Graphical Abstract:**
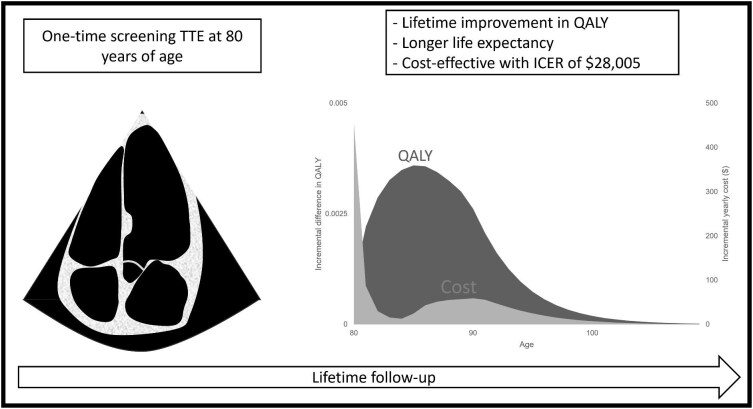

**Abbreviations**
AS: aortic stenosis
AV: aortic valve
TTE: transthoracic echocardiogram
TAVR: transcatheter aortic valve replacement
ICER: cost-effectiveness ratio
NMB: net monetary benefit

## Introduction

There is a high burden of asymptomatic valvular heart disease in the general population, with a prevalence directly related to age.^1–4^ Degenerative aortic stenosis (AS) is the most common valvular disease in the Western world, representing a substantial and increasing disease burden in the ageing population.^1,5^ In adults ≥75 years of age, AS is present in 12.4% of individuals and severe AS in 3.4%. In 2020, over 43 million adults ≥75 years of age were living in Europe, with approximately 5.4 million adults with AS and 1.5 million with severe AS. The EU population is projected to increase to over 75 million by 2050, with an expected 9.3 million adults with AS and 2.6 million with severe AS.^6^ Untreated, symptomatic severe AS is associated with significant morbidity and mortality, with a 1-year all-cause mortality above 50%.^7^ In the modern era of structural heart disease, transcatheter aortic valve replacement (TAVR) has been demonstrated to be a minimally invasive and cost-effective alternative to cardiac surgery.^8,9^ With the excellent safety and efficacy profile of TAVR, its use has rapidly expanded to become the near exclusive modality for AVR in elderly patients ≥80 years of age, as well as receiving a class I indication for the treatment of symptomatic AS in patients 65 years of age and older.^10,11^

The significant disease burden of AS has prompted calls for screening campaigns for early detection.^12–14^ Screening methods including auscultation, artificial intelligence algorithms, and bedside ultrasound have been previously studied.^15,16^ While these remain as attractive alternatives, screening with comprehensive transthoracic echocardiography (TTE) is a relatively inexpensive test and has comparable cost to other screening modalities.^11,17,18^ While not commonly seen with current screening programs, the low cost of TTE allows for screening with the diagnostic test.

Current studies screening for valvular heart disease focus on the prevalence of undiagnosed disease. While this is an important aspect of screening, the benefit of screening relies on a holistic assessment of the economic impact of these programs. The World Health Organization (WHO) recommends that screening programs be economically balanced with inclusion of the costs associated with early diagnosis, interventions, and follow-up.^19^ In the absence of disease modifying or early intervention strategies, the economic benefit and patient outcomes with screening in the prevalent geriatric population are uncertain.^20^

The objective of this study is to determine the cost-effectiveness of AS screening in the geriatric population using the diagnostic standard TTE. The purpose is to identify undiagnosed severe AS, as well as diagnosing early AS to facilitate timely intervention at symptom onset. As part of the economic evaluation, screening at 70 and 80 years of age was performed to determine the most cost-effective timing for screening.

## Methods

### Model

A Markov model was developed using good modelling practices to estimate the cost-effectiveness ratio (ICER) and net monetary benefit (NMB) for AS screening with a one-time TTE.^21^ Mutually exclusive health states for diagnosed and undiagnosed AS were included, along with post-TAVR health states. The setting for screening is at the level of primary care, and the perspective is that of the publicly funded Canadian Healthcare system.

**Figure 1:**
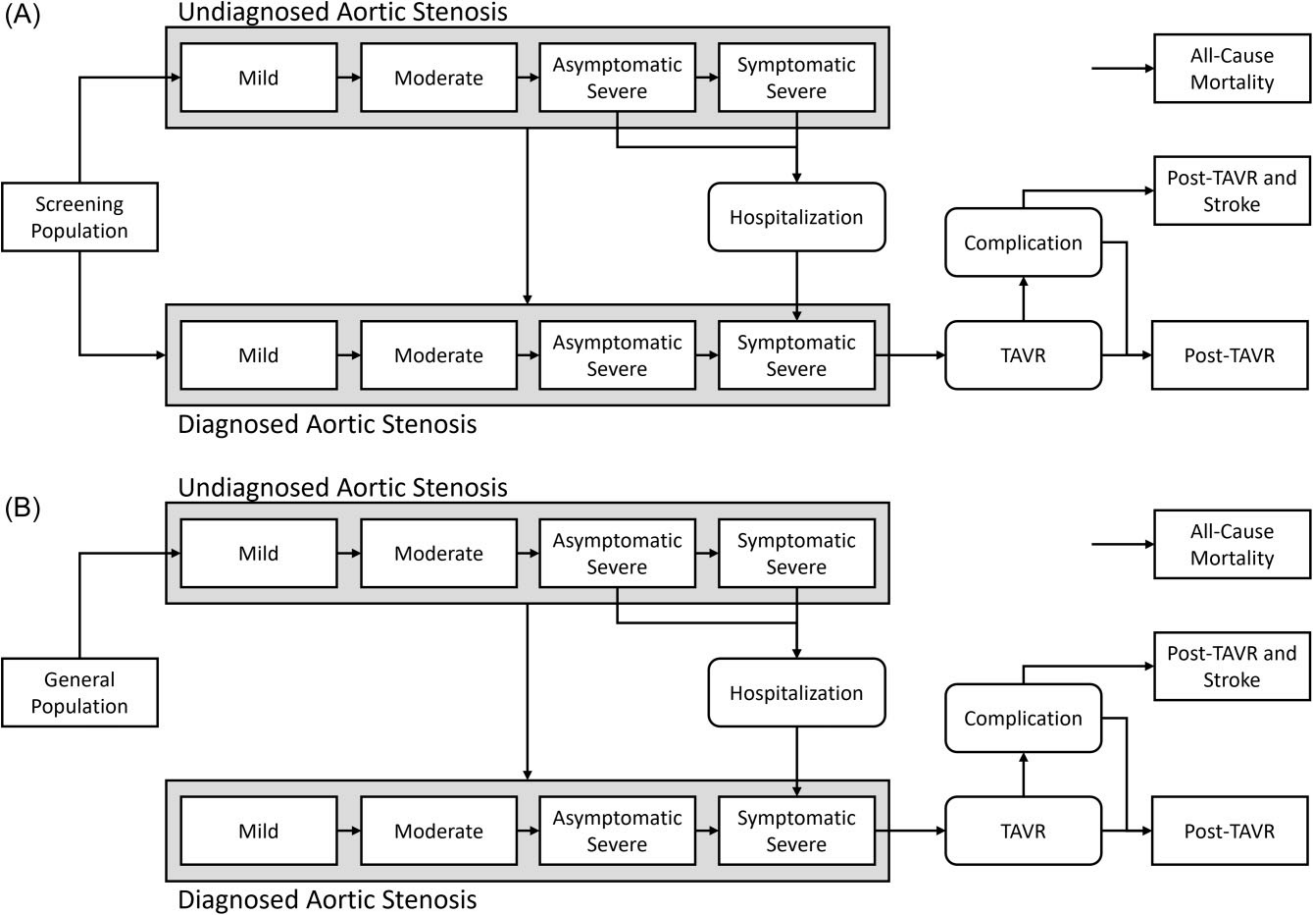
Basic description of the structure in the decision analytic Markov model. (*A*) One-time screening model and (*B*) Standard of care—non-screening model. The post-TAVR complications include stroke, major non-cerebral bleed, vascular complications, and pacemaker implantation. As the impact on quality of life with non-stroke complications are transient, these patients will transition to the post-TAVR health state. The post-TAVR with stroke state was separated to include the longer lasting costs and disutility of stoke. TAVR, transcatheter aortic valve replacement.

The screening and non-screening models, the latter being the current standard of care, are highlighted in *Figure 1*. In the screening model, a one-time screening TTE will have patients either transition to the diagnosed AS health states or remain in the screening/general population. As patients can develop AS after their screening TTE, patients can transition from the screening population to undiagnosed AS. AS severity was defined by aortic valve area, where mild, moderate, and severe disease were defined as >1.5 cm^2^, 1.0–1.5 cm^2^, and <1 cm^2^, respectively.^22^ In the undiagnosed AS health states, patients can progress in severity and/or transition to diagnosed AS. Symptomatic severe AS can be diagnosed by an index hospitalization. Once diagnosed with severe symptomatic AS, patients will undergo TAVR in the following cycle. The post-TAVR complications––stroke, major non-cerebral bleed, vascular complications, and pacemaker implantation––were included. As the impact on quality of life with non-stroke complications is transient, these patients will transition to the post-TAVR health state. The post-TAVR with stroke state was separated to include the longer lasting costs and disutility of stroke; these post-stroke health states are tunnelled to account for the difference in cost and disutility before and after 1 year. All patients can transition to death from any health state. In the non-screening model, all AS will be undiagnosed before transitioning to the diagnosed health states.

The following assumptions were made during model development: (i) all patients are medically and anatomically suitable for TAVR, (ii) all patients undergo TAVR and not surgical AVR,^23^ (iii) as current outcome studies for TAVR are limited to 5-year data and the longevity of TAVR valves remain undefined, no repeat procedures were included, and (iv) of those who have a complication, only one would occur.

### Population

Two screening populations were included for the primary analysis: (i) patients without known AS at 70 years of age, and (ii) patients without known AS at 80 years of age.

### Aortic stenosis screening

For the primary analysis of screening at 70 years of age, the OxValve Population Cohort Study (OxValve PCS) was used to determine the prevalence of AS.^3^ This study screened 2500 individuals 65 years of age or older with TTE and found a prevalence of 35.3% for calcific aortic valve disease, with AS present in 1.3% of the total population. Of all AS cases, 98% had mild disease, with the remaining having moderate disease. The BELFRAIL study was used for screening at 80 years of age. This cross-sectional study included 556 individuals aged 80 years or older who underwent screening examinations and TTE. Of the total cohort, 37 had mild AS, 57 had moderate AS, and 33 had severe AS^2^ (*Table 1*).

**Table 1:** Screening probabilities


	Screen positive rate	Distribution	Undiagnosed mild AS	Undiagnosed moderate AS	Undiagnosed severe AS	Diagnosed mild AS	Diagnosed moderate AS	Diagnosed severe AS	Distribution	Reference
Screening at 70 years of age										
Screening	0.013	Beta	0	0	0	0.981	*	0	Beta	^3^
No screening	-	-	0.981	*	0	0	0	0	Beta	
Screening at 80 years of age										
Screening	0.228	Beta	0	0	0	0.300	0.429	0.271	Gamma	^2^
No screening	-	-	0.300	0.429	0.271	0	0	0	Gamma	

AS, aortic stenosis.

* Remaining probability after accounting for mild AS.

For both models, the transition from the screening population to undiagnosed AS after screening TTE was calibrated based on the prevalence of aortic sclerosis due to its association with AS development.^3,24^

### Transition probabilities

The per-cycle (monthly) transition probabilities are shown in *Table 2*. Prospective data from a systematic review and meta-analysis evaluating the rates of AS progression according to baseline severity was used.^25^ In the case of progression from severe asymptomatic to symptomatic AS, natural history studies were used.^1^ Transition probabilities from undiagnosed to diagnosed AS health states were based on a Canadian database study; as non-severe AS individuals are asymptomatic, the average utilization rate of 4.5% per year was used.^26^ In the case of undiagnosed symptomatic severe AS, the average time from first symptom onset to diagnosis was estimated to be 6–12 months based on expert consensus. The diagnosis of severe AS through an index admission was determined using Ontario registry data of the trends of AS hospitalization.^27^

**Table 2:** Transition probabilities

Probability	Input	Probability distribution	Reference
General population To death To undiagnosed mild AS^a^	Age specific mortality 0.0122	Beta Beta	^41^ ^3,24^
AVA progression (standard error)	0.07 (0.0128)	Gamma	^25^
Mild aortic stenosis To moderate aortic stenosis To death^b^ Undiagnosed to diagnosed	Age specific mortality 0.0037	- Beta Beta	- ^42^ ^26^
Moderate aortic stenosis To severe aortic stenosis To death^b^ Undiagnosed to diagnosed	* Age specific mortality 0.0037	- Beta Beta	- ^42^ ^26^
Severe asymptomatic aortic stenosis To severe symptomatic aortic stenosis To death^c^ Undiagnosed to diagnosed (without hospitalization) Undiagnosed to diagnosed (with index hospitalization)	0.0153 Age specific mortality 0.0037 0.0046	Beta Beta Beta Beta	^1^ ^42–44^ ^26^ ^45^
Severe symptomatic aortic stenosis to death^c^	0.0305	Beta	^42–44^
TAVR No complications Complication: stroke Complication: PPM insertion Complication: major bleeding Complication: vascular complication Death	^**^ 0.022 0.116 0.076 0.093 0.026	Fixed Beta Beta Beta Beta Beta	^46^ ^46^ ^47^ ^47^ ^46^
Post-TAVR with stroke (relative risk)^d^ Year one to death Year two onwards to death	4.46 1.99	Lognormal Lognormal	^48^ ^48^

a Calibrated on the prevalence of aortic sclerosis.

b Calibrated on the relative risk of death in comparison to general population.

c Calibrated on the relative risk of death in comparison to general population and prevalence of symptomatic severe aortic stenosis.

d Calibrated on the relative risk of death in comparison to general population.

* Derived from AVA progression.

** Remaining probability after accounting for complications.

TAVR, transcatheter aortic valve implantation.

In terms of complication rates post-TAVR, registry data was preferred over the published randomized controlled trials, as the latter are confounded by patient risk and temporal changes in TAVR technique.

Canadian age-adjusted mortality rates were used for this study. Mild, moderate, and severe AS mortality probabilities were calibrated based on severity specific relative risks of mortality. In the post-TAVR health states, mortality rate returns to the respective age-adjusted mortality unless the procedure was complicated by stroke.

### Cost and utilities

In the screening model, a one-time TTE fee was applied in the first cycle. In both models, the costs associated with diagnosed AS were based on the American Heart Association valvular disease guidelines.^11^ A TTE would be completed every 4 years (recommendation every 3–5 years in guidelines) in diagnosed mild AS, every 18 months (recommendation every 1–2 years) with a cardiology assessment in diagnosed moderate AS, and every 9 months (recommendation every 6–12 months) in those with severe asymptomatic AS. The cost of index hospitalization was calculated with unpublished patient-level data provided from Canadian Institute of Health Information (CIHI). The costs associated with TAVR including the cost of the device, physician and procedural fees, hospitalization, and pre-procedure testing have been previously published^28^ (*Table 3*).

**Table 3:** Cost and utility input values

Parameter	Base estimate	Probability distribution	Reference
*Utility*			
Screening population	0.83	Lognormal	^49^
Aortic stenosis Mild Moderate Severe asymptomatic Severe symptomatic	0.83 0.83 0.83 0.57	Lognormal Lognormal Lognormal Lognormal	^49^ ^49^ ^49^ ^50^
Post-TAVR without complications	0.83	Lognormal	^49^
Post-TAVR with non-stroke complication	0.83	Lognormal	^49^
Post-TAVR with stroke (year 1)	0.64	Lognormal	^30^
Post-TAVR with stroke (year 2 onwards)	0.69	Lognormal	^29^
*Costs*			
Cost of screening (one-time cost)	$215.15	Fixed	^51^ G570, G571
Diagnosed AS (per monthly cycle) Mild Moderate Severe	$4.48 $11.95 $41.35	Gamma Gamma Gamma	^11,51^ ^11,51^ ^11,51^
Index hospitalization (one-time cost)	$7940	Gamma	^52^, CIHI 195
TAVR (one-time cost)	$64 466	Gamma	^28^
Non-stroke complications Pacemaker implantation Vascular complication Major bleeding	$11 209 $12894.98 $3428.16	Gamma Gamma Gamma	^52^ CIHI 187 ^53^ CMG + 213 ^53^ CMG + 782
Post-TAVR—one time follow-up	$105.25	Fixed	^51^ A606
Post-TAVR with stroke (year 1—per monthly cycle)^a^	$6196.08	Gamma	^54^
Post-TAVR with stroke (year 2 onwards—per monthly cycle)	$522.08	Gamma	^55^

AS, aortic stenosis; TAVR, transcatheter aortic valve implantation.

a Includes cost of index event.

Utility of mild and moderate AS, and non-stroke post-TAVR health states was assumed to be the same as the screening population, as these groups are overall asymptomatic from their disease. Severe disease utility is based on primary data from the PARTNER trials.^28^ Post-TAVR with stroke utility was derived from a systematic review and meta-analysis of stroke utility (*Table 1*).^29,30^ There was no disutility with hospitalization or non-stroke complications, as these were assumed to be transient in nature. No adjustments were made to the extracted utility values.

### Analysis

**Figure 2:**
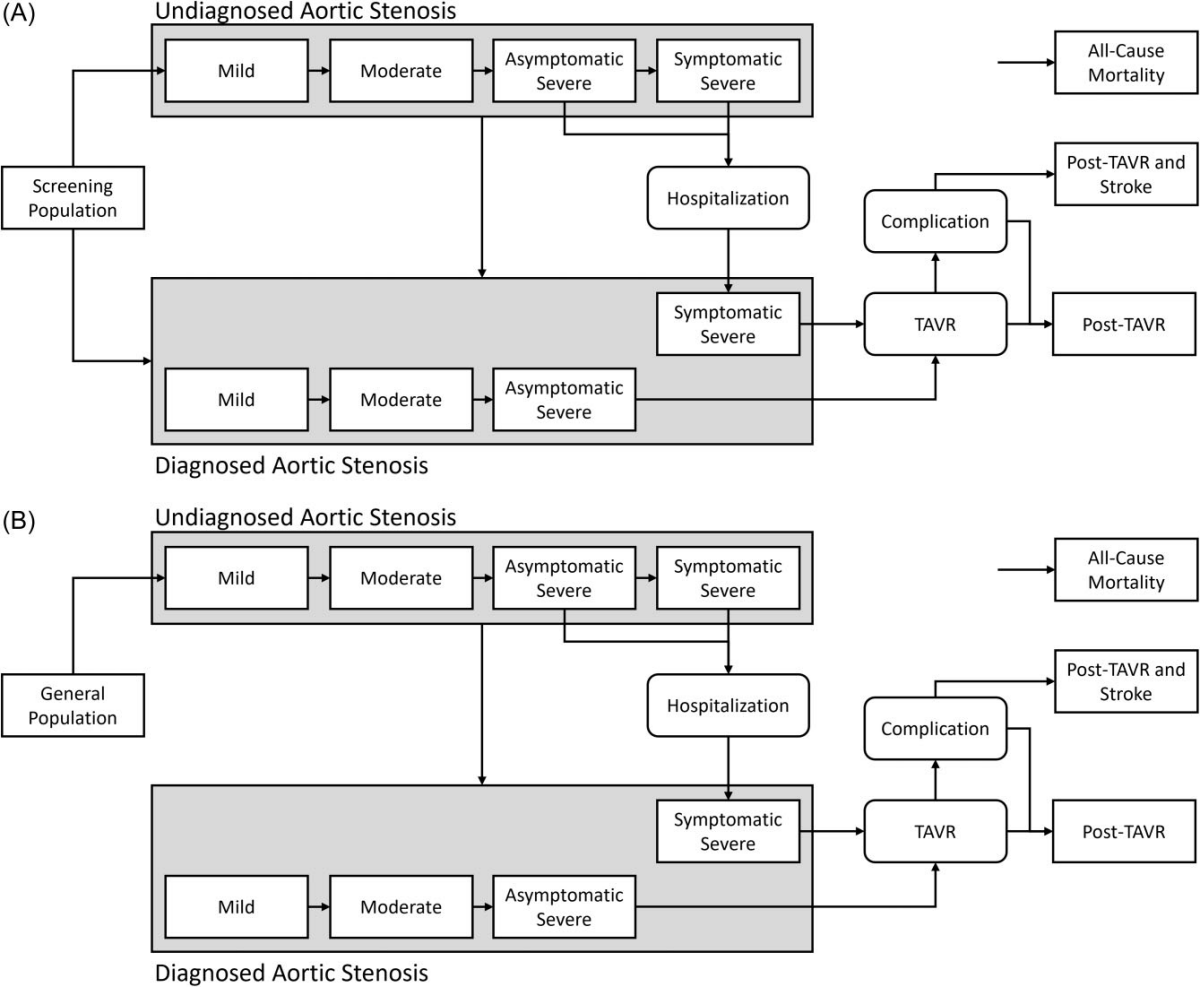
Basic description of the structure in the decision analytic Markov model for early intervention. (*A*) One-time screening model and (*B*) Standard of care—non-screening model. The post-TAVR complications include stroke, major non-cerebral bleed, vascular complications, and pacemaker implantation. As the impact on quality of life with non-stroke complications are transient, these patients will transition to the post-TAVR health state. The post-TAVR with stroke state was separated to include the longer lasting costs and disutility of stoke. TAVR, transcatheter aortic valve replacement.

Probabilistic sensitivity analysis using a Monte Carlo simulation with 5000 replications was used to calculate an ICER and NMB for both screening methods. Scenario analysis with TAVR at the time of severe AS diagnosis, irrespective of symptoms, was also completed (*Figure 2*). Data analysis was completed with a lifetime horizon with a 1.5% discount rate for costs and outcomes. The cycle length was 1-month to reduce the risk of progression over multiple health states.

## Results

### Primary case-base analysis: TAVR in severe symptomatic aortic stenosis

The findings of the probabilistic analysis are summarized in *Table 4*. In patients 70 years of age, screening for AS is associated with a lifetime QALY of 12.81. There is a minimal increase of 0.0015 in comparison to the non-screening model. There is an incremental increase in the cost of screening of $242 ($34 518 compared to $34 760). The respective ICER is $156 722 and the NMB is −$165 when willingness-to-pay is set at $50 000 per QALY, making it not cost-effective to screen patients at 70 years of age.

**Table 4:** Primary case base analysis results (severe symptomatic)

Probabilistic result	Screening	No screening	Incremental change
*Screening at 70 years of age*			
QALY	12.81	12.81	0.00
Costs	$34 760	$34 518	$242
		ICER	$156 722
		Net benefit (QALY = $50 000)	−$165
*Screening at 80 years of age*			
QALY	7.37	7.33	0.05
Costs	$24 141	$22 854	$1287
		ICER	$28 005
		Net benefit (QALY = $50 000)	$1011

**Figure 3:**
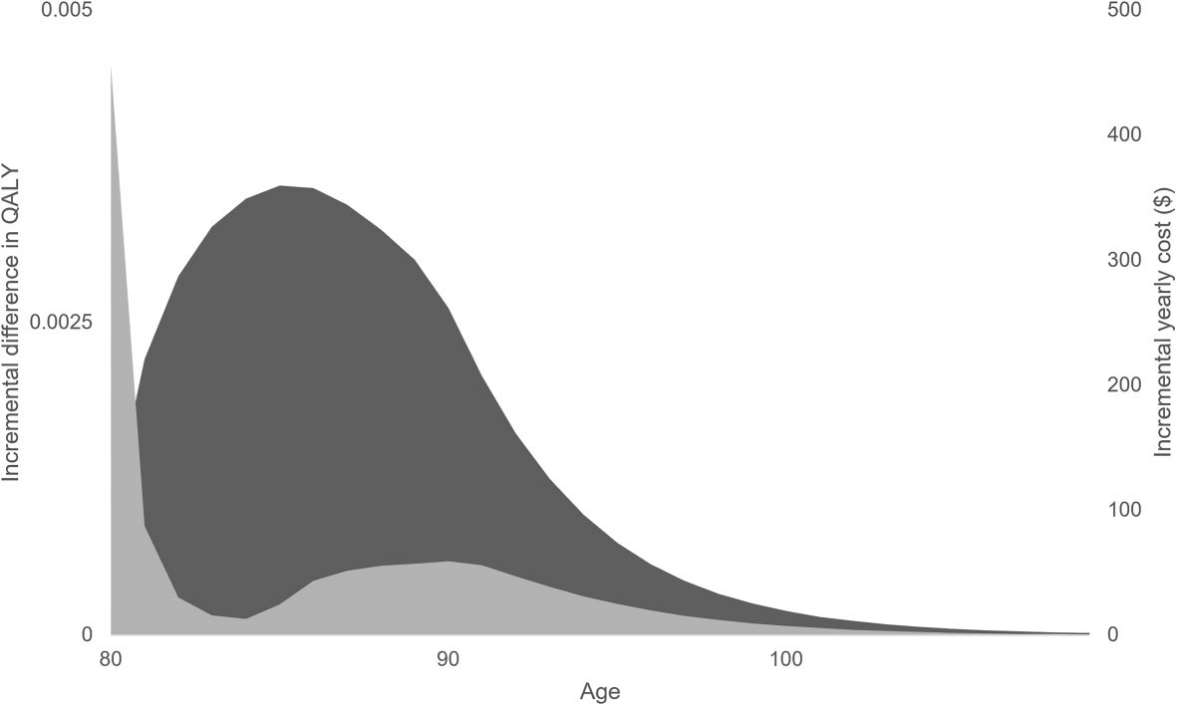
Probabilistic Analysis using a Monte Carlo Simulation of 5000 replications on the incremental gain in QALY (black; primary vertical axis) and monthly cost (grey; secondary vertical axis) with screening at 80 years of age over a lifetime horizon.

In patients 80 years of age, screening for AS yielded a QALY of 7.37 in comparison to 7.33 with the non-screening model. The incremental QALY and cost with screening are 0.05 and $1287, making for an ICER of $28 005. Using a willingness-to-pay of $50 000 per QALY, screening at 80 years of age is cost-effective from a public health perspective with a NMB of $1011. *Figure 3* demonstrates the incremental yearly cost and QALY when screening at age 80 years. QALY is incrementally greater with screening over the lifetime horizon, with the difference predominantly over the 80 s.

### Scenario case-base analysis: TAVR in severe aortic stenosis

Results of the scenario case-base analysis for early TAVR for asymptomatic severe AS are summarized in *Table 5*. In patients screened at 70 years of age, there is a minimal improvement in QALY by 0.01 that is associated with an increase in cost by $1012. The ICER is $142 157 with a NMB of −$656, making it not cost-effective to screen for severe AS in patients 70 years of age when early TAVR is performed in asymptomatic severe AS patients.

**Table 5:** Scenario case base analysis results for early intervention (severe asymptomatic)

Probabilistic result	Screening	No screening	Incremental change
*Screening at 70 years of age*			
QALY	11.99	11.99	0.01
Costs	$21 399	$20 388	$1012
		ICER	$142 157
		Net benefit (QALY = $50 000)	−$656
*Screening at 80 years of age*			
QALY	7.36	7.12	0.24
Costs	$44 388	$14 387	$30 001
		ICER	$124 651
		Net benefit (QALY = $50 000)	−$17 967

In patients screened at 80 years of age, screening for AS with an early intervention in asymptomatic severe AS patients was not cost-effective, with an ICER of $124 651 and an NMB of −$17 967. There is an increase in QALY of 0.24, but also a cost increase of $30 001, making it not cost-effective to screen for AS in patients 80 years of age when early TAVR is performed in asymptomatic severe patients.

## Discussion

In this first health-economic study evaluating the cost-effectiveness of screening for AS in the geriatric population, we found that screening at 80 years of age was cost-effective from a public health perspective with the current management strategy of performing TAVR when patients become symptomatic. Earlier screening at 70 years of age, or screening for AS at 70 or 80 years of age and employing a strategy of early intervention on asymptomatic patients with severe AS, was not cost-effective based on the willingness-to-pay of $50 000 per QALY.

The geriatric population is often unrepresented in clinical studies and health care policies.^31^ Current screening recommendations traditionally exclude geriatric patients due to minimal gain in benefit from competing risks of morbidity and mortality.^32^ However, AS primarily impacts older patients and has a significant disease burden due to the high prevalence and its associated morbidity and mortality.^4,33^ As a result, recent reviews have highlighted that valvular heart disease is often under-detected and diagnosed late, calling for efforts to improve community awareness and evaluate screening programs.^13^ In response, screening modalities have been developed, including the use of deep learning AI algorithms to detect valvular heart disease on precordial auscultation, bedside ultrasound, and AI-integrated electrocardiograms.^34–36^ While these are attractive alternatives, a diagnostic comprehensive TTE is relatively inexpensive and highly accurate in diagnosing and quantifying disease severity compared to other screening modalities.^17,18^ Furthermore, these alternative screening modalities require additional training, equipment, and personnel and may not be highly sensitive or specific, making these modalities less feasible in real-world clinical practice. With established infrastructure and expertise in TTE widely available, an AS screening program becomes a feasible method to incorporate screening broadly.

In our study, individuals 80 years of age who undergo TAVR based on the presence of symptoms are cost-effective from a public health perspective as it mitigates the costs, morbidity, and mortality associated with the diagnostic and referral delays in patients with severe AS.^37,38^ Screening was not cost-effective in individuals 70 years of age when TAVR was performed after the development of symptoms, which is likely due to the lower prevalence of AS in younger individuals, lower incidence rate of severe AS, and the increased duration and cost of follow-up.^1^ In the scenario analysis, screening at 80 years of age with a management strategy of early intervention at the time of diagnosis of severe asymptomatic AS was not as cost-effective. This remains counterintuitive when compared with other screening programs, where the emphasis is often on intervening while the patient is asymptomatic. This finding is a reflection of the geriatric population. While the presence of AS, irrespective of severity or symptoms, impacts mortality, early intervention is not cost-effective owing to the competing risk of death from other diseases such as cancer and stroke in this age cohort.^39^ In a Japanese cohort of patients with severe AS managed conservatively, 44% of patients had a non-cardiac cause of death, while 46% of patients managed with AVR had a non-cardiac cause of death.^40^ Our analysis suggests that the benefit of screening is a function of timely intervention in the presence and onset of symptoms rather than early intervention. This finding is critically important in the context of ongoing randomized clinical trials, including the Evaluation of TAVR Compared to Surveillance for Patients with Asymptomatic Severe Aortic Stenosis (NCT03042104) and the Prospective, Randomized, Controlled Trial to Assess the Management of Moderate Aortic Stenosis by Clinical Surveillance or Transcatheter Aortic Valve Replacement (NCT04889872). Whether these trials identify clinical benefit in early TAVR for asymptomatic AS, our data suggest that on a population level, screening for AS to identify these patients lacks cost effectiveness.

According to the World Health Organization Principles and Practices of Screening for Disease, AS is an important health problem with a well-established natural history and an early symptomatic stage whereby diagnosis with comprehensive TTE is accurate and sensitive and TAVR is a well-established and effective treatment.^19^ Additionally, from a public health perspective, screening must also be cost-effective. The results of this study support the cost effectiveness aspect of disease screening.

This study is not without limitations. First, the cost-effectiveness of screening for AS is directly related to the prevalence of undiagnosed disease. While there is a systematic review and meta-analysis on the prevalence of AS in the geriatric population, the geographic and age-specific prevalence are limited to the individual studies used in our analysis.^1^ Additionally, sex-related differences exist in the age of diagnosis and timing of intervention, as well as the rates of TAVR use, which we could not account for based on the prevalence data.^23^ The development of AS screening programs should therefore be preceded by the generation of regionally relevant data on age and sex-related disease prevalence. Second, while we have identified a screening age, there needs to be further consensus on important inclusion and exclusion criteria for screening. For example, critical frailty, life-limiting non-cardiac disease, and patient preference should be considered prior to screening. Furthermore, a proportion of the population may have had a previous TTE, and the time interval in which a repeat TTE would not be warranted is unclear. Third, during model development, we made assumptions that patients would not undergo surgical management (TAVR performed in 97.8% of patients >80 years of age^23^) and that no repeat valve-in-valve TAVR would be required for the remainder of life.^28^ Lastly, a screening comprehensive TTE would potentially identify other cardiac disorders, including other forms of valvular heart disease or cardiomyopathies. However, the impact of this issue is likely low, as highlighted by the BELFRAIL study, where only one patient was found to have another severe valvulopathy.^2^

The implication of this study is that population-based screening for AS in individuals 80 years of age in Canada is cost-effective when TAVR is performed in severe AS based on the presence of symptoms. Further research should be focused on identifying the geographical differences in AS prevalence to further elucidate the cost effectiveness of AS screening programs.

## Data Availability

No new data were generated or analysed in support of this research.

## References

[1] Osnabrugge RLJ, Mylotte D, Head SJ, Van Mieghem NM, Nkomo VT, Lereun CM et al. Aortic stenosis in the elderly: disease prevalence and number of candidates for transcatheter Aortic valve replacement: a meta-analysis and modeling study. J Am Coll Cardiol 2013;62:1002–1012. 10.1016/j.jacc.2013.05.015 23727214

[2] Vaes B, Rezzoug N, Pasquet A, Wallemacq P, Van Pottelbergh G, Matheï C et al. The prevalence of cardiac dysfunction and the correlation with poor functioning among the very elderly. Int J Cardiol 2012;155:134–143. 10.1016/j.ijcard.2011.07.024 21839531

[3] D'arcy JL, Coffey S, Loudon MA, Kennedy A, Pearson-Stuttard J, Birks J et al. Large-scale community echocardiographic screening reveals a major burden of undiagnosed valvular heart disease in older people: the OxVALVE Population Cohort Study†. Eur Heart J 2016;37:3515–3522. 10.1093/eurheartj/ehw229 27354049 PMC5216199

[4] Nkomo VT, Gardin JM, Skelton TN, Gottdiener JS, Scott CG, Enriquez-Sarano M. Burden of valvular heart diseases: a population-based study. Lancet North Am Ed 2006;368:1005–1011. 10.1016/S0140-6736(06)69208-8 16980116

[5] Bonow RO, Greenland P. Population-wide trends in aortic stenosis incidence and outcomes. Circulation 2015;131:969–971. 10.1161/circulationaha.115.014846 25691712

[6] EUROSTAT 2020. ec.europa.eu/eurostat/data/database (November 27, 2022)

[7] Leon MB, Smith CR, Mack M, Miller DC, Moses JW, Svensson LG et al. Transcatheter aortic-valve implantation for aortic stenosis in patients who cannot undergo surgery. N Engl J Med 2010;363:1597–1607. 10.1056/NEJMoa1008232 20961243

[8] Zhou JY, Liew D, Duffy SJ, Walton A, Htun N, Stub D. Cost-effectiveness of transcatheter versus surgical aortic valve replacement in low-risk patients with severe aortic stenosis. Heart, Lung Circ 2021;30:547–554. 10.1016/j.hlc.2020.09.934 33189571

[9] Baron SJ, Wang K, House JA, Magnuson EA, Reynolds MR, Makkar R et al. Cost-effectiveness of transcatheter versus surgical aortic valve replacement in patients with severe aortic stenosis at intermediate risk. Circulation 2019;139:877–888. 10.1161/CIRCULATIONAHA.118.035236 30586747

[10] Prosperi-Porta G, Nguyen V, Willner N, Dreyfus J, Eltchaninoff H, Burwash IG et al. Association of age and sex with use of transcatheter aortic valve replacement in France. J Am Coll Cardiol 2023. 10.1016/j.jacc.2023.08.044 37877906

[11] Otto CM, Nishimura RA, Bonow RO, Carabello BA, Erwin JP, Gentile F et al. 2020 ACC/AHA Guideline for the management of patients with valvular Heart disease: a report of the American College of Cardiology/American Heart Association Joint Committee on Clinical Practice Guidelines. Circulation 2021;143:e72–e227. 10.1161/CIR.0000000000000923 33332150

[12] Thoenes M, Bramlage P, Zamorano P, Messika-Zeitoun D, Wendt D, Kasel M et al. Patient screening for early detection of aortic stenosis (AS)-review of current practice and future perspectives. J Thorac Dis 2018;10:5584–5594. 10.21037/jtd.2018.09.02 30416809 PMC6196210

[13] Messika-Zeitoun D, Baumgartner H, Burwash IG, Vahanian A, Bax J, Pibarot P et al. Unmet needs in valvular heart disease. Eur Heart J 2023:ehad121. 10.1093/eurheartj/ehad121 36924203

[14] Steeds RP, Potter A, Mangat N, Fröhlich M, Deutsch C, Bramlage P et al. Community-based aortic stenosis detection: clinical and echocardiographic screening during influenza vaccination. Open Heart 2021;8:e001640. 10.1136/openhrt-2021-001640 34021069 PMC8144056

[15] Cohen-Shelly M, Attia ZI, Friedman PA, Ito S, Essayagh BA, Ko W-Y et al. Electrocardiogram screening for aortic valve stenosis using artificial intelligence. Eur Heart J 2021;42:2885–2896. 10.1093/eurheartj/ehab153 33748852

[16] Makimoto H, Shiraga T, Kohlmann B, Magnisali CE, Gerguri S, Motoyama N et al. Efficient screening for severe aortic valve stenosis using understandable artificial intelligence: a prospective diagnostic accuracy study. European Heart Journal—Digital Health 2022;3:141–152. 10.1093/ehjdh/ztac029 36713014 PMC9707975

[17] Feig S . Cost-effectiveness of mammography, MRI, and ultrasonography for breast cancer screening. Radiologic Clinics 2010;48:879–891. 10.1016/j.rcl.2010.06.002 20868891

[18] Sonnenberg A, Delcò F, Inadomi JM. Cost-effectiveness of colonoscopy in screening for colorectal cancer. Ann Intern Med 2000;133:573–584. 10.7326/0003-4819-133-8-200010170-00007 11033584

[19] Wilson JMG, Jungner G, Organization WH. Principles and practice of screening for disease. 1968. doi:

[20] Marquis-Gravel G, Redfors B, Leon MB, Généreux P. Medical treatment of aortic stenosis. Circulation 2016;134:1766–1784. 10.1161/CIRCULATIONAHA.116.023997 27895025

[21] Siebert U, Alagoz O, Bayoumi AM, Jahn B, Owens DK, Cohen DJ et al. State-transition modeling: a report of the ISPOR-SMDM Modeling Good Research Practices Task Force—3. Value Health 2012;15:812–820. 10.1016/j.jval.2012.06.014 22999130

[22] Baumgartner H, Hung J, Bermejo J, Chambers JB, Edvardsen T, Goldstein S et al. Recommendations on the echocardiographic assessment of aortic valve stenosis: a focused update from the European Association of Cardiovascular Imaging and the American Society of Echocardiography. European Heart Journal-Cardiovascular Imaging 2017;18:254–275. doi:28363204 10.1093/ehjci/jew335

[23] Prosperi-Porta G, Nguyen V, Willner N, Dreyfus J, Eltchaninoff H, Burwash IG et al. Association of age and sex with use of transcatheter aortic valve replacement in France. J Am Coll Cardiol 2023;82:1889–1902. 10.1016/j.jacc.2023.08.044 37877906

[24] Olsen MH, Wachtell K, Bella JN, Liu JE, Boman K, Gerdts E et al. Effect of losartan versus atenolol on aortic valve sclerosis (a LIFE substudy). Am J Cardiol 2004;94:1076–1080. 10.1016/j.amjcard.2004.06.074 15476632

[25] Willner N, Prosperi-Porta G, Lau L, Nam Fu AY, Boczar K, Poulin A et al. Aortic stenosis progression: a systematic review and meta-analysis. JACC Cardiovasc Imaging 2023;16:314–328. 10.1016/j.jcmg.2022.10.009 36648053

[26] Blecker S, Bhatia RS, You JJ, Lee DS, Alter DA, Wang JT et al. Temporal trends in the utilization of echocardiography in Ontario, 2001 to 2009. JACC Cardiovasc Imaging 2013;6:515–522. 10.1016/j.jcmg.2012.10.026 23579013 PMC3915739

[27] Czarnecki A, Qiu F, Koh M, Alter DA, Austin PC, Fremes SE et al. Trends in the incidence and outcomes of patients with aortic stenosis hospitalization. Am Heart J 2018;199:144–149. 10.1016/j.ahj.2018.02.010 29754653

[28] Tarride J-E, Luong T, Goodall G, Burke N, Blackhouse G. A Canadian cost-effectiveness analysis of SAPIEN 3 transcatheter aortic valve implantation compared with surgery, in intermediate and high-risk severe aortic stenosis patients. Clinicoecon Outcomes Res 2019;11:477–486. 10.2147/ceor.S208107 31551658 PMC6677373

[29] Joundi RA, Adekanye J, Leung AA, Ronksley P, Smith EE, Rebchuk AD et al. Health State utility values in people with stroke: a systematic review and meta-analysis. J Am Heart Assoc 2022;11:e024296. 10.1161/jaha.121.024296 35730598 PMC9333363

[30] Betts MB, Rane P, Bergrath E, Chitnis M, Bhutani MK, Gulea C et al. Utility value estimates in cardiovascular disease and the effect of changing elicitation methods: a systematic literature review. Health Qual Life Outcomes 2020;18:251. 10.1186/s12955-020-01407-y 32718355 PMC7385861

[31] Hutchins LF, Unger JM, Crowley JJ, Coltman CA, Albain KS. Underrepresentation of patients 65 years of age or older in cancer-treatment trials. N Engl J Med 1999;341:2061–2067. 10.1056/nejm199912303412706 10615079

[32] Leddin D, Hunt R, Champion M, Cockeram A, Flook N, Gould M et al. Canadian Association of Gastroenterology and the Canadian Digestive Health Foundation: guidelines on colon cancer screening. Can J Gastroenterol 2004;18:93–99. doi:14997217 10.1155/2004/983459

[33] Coffey S, Roberts-Thomson R, Brown A, Carapetis J, Chen M, Enriquez-Sarano M et al. Global epidemiology of valvular heart disease. Nat Rev Cardiol 2021;18:853–864. 10.1038/s41569-021-00570-z 34172950

[34] Chorba JS, Shapiro AM, Le L, Maidens J, Prince J, Pham S et al. Deep learning algorithm for automated cardiac murmur detection via a digital stethoscope platform. J Am Heart Assoc 2021;10:e019905. 10.1161/JAHA.120.019905 33899504 PMC8200722

[35] Narang A, Bae R, Hong H, Thomas Y, Surette S, Cadieu C et al. Utility of a deep-learning algorithm to guide novices to acquire echocardiograms for limited diagnostic use. JAMA Cardiol 2021;6:624–632. 10.1001/jamacardio.2021.0185 33599681 PMC8204203

[36] Cohen-Shelly M, Attia ZI, Friedman PA, Ito S, Essayagh BA, Ko W-Y et al. Electrocardiogram screening for aortic valve stenosis using artificial intelligence. Eur Heart J 2021;42:2885–2896. 10.1093/eurheartj/ehab153 33748852

[37] Albassam O, Henning KA, Qiu F, Cram P, Sheth TN, Ko DT et al. Increasing wait-time mortality for severe aortic stenosis: a population-level study of the transition in practice from surgical aortic valve replacement to transcatheter aortic valve replacement. Circ Cardiovasc Interv 2020;13:e009297. 10.1161/circinterventions.120.009297 33167700

[38] Gjertsson P, Caidahl K, Odén A, Bech-Hanssen O. Diagnostic and referral delay in patients with aortic stenosis is common and negatively affects outcome. Scand Cardiovasc J 2007;41:12–18. 10.1080/14017430601115935 17365972

[39] Brooke HL, Talbäck M, Hörnblad J, Johansson LA, Ludvigsson JF, Druid H et al. The Swedish cause of death register. Eur J Epidemiol 2017;32:765–773. 10.1007/s10654-017-0316-1 28983736 PMC5662659

[40] Minamino-Muta E, Kato T, Morimoto T, Taniguchi T, Shiomi H, Nakatsuma K et al. Causes of death in patients with severe aortic stenosis: an observational study. Sci Rep 2017;7:14723. 10.1038/s41598-017-15316-6 29116212 PMC5676690

[41] Canada S . Mortality rates, by age group. doi:

[42] Stewart S, Afoakwah C, Chan Y-K, Strom JB, Playford D, Strange GA. Counting the cost of premature mortality with progressively worse aortic stenosis in Australia: a clinical cohort study. Lancet Healthy Longev 2022;3:e599–e606. 10.1016/s2666-7568(22)00168-4 36102774 PMC9484033

[43] Gahl B, Çelik M, Head SJ, Vanoverschelde J-L, Pibarot P, Reardon MJ et al. Natural History of asymptomatic severe Aortic Stenosis and the Association of Early Intervention with Outcomes: a systematic review and meta-analysis. JAMA Cardiol 2020;5:1102–1112. 10.1001/jamacardio.2020.2497 32639521 PMC7344834

[44] Osnabrugge RLJ, Mylotte D, Head SJ, Van Mieghem NM, Nkomo VT, Lereun CM et al. Aortic stenosis in the elderly: disease prevalence and number of candidates for transcatheter aortic valve replacement: a meta-analysis and modeling study. J Am Coll Cardiol 2013;62:1002–1012. 10.1016/j.jacc.2013.05.015 23727214

[45] Kanamori N, Taniguchi T, Morimoto T, Shiomi H, Ando K, Murata K et al. Asymptomatic versus symptomatic patients with severe aortic stenosis. Sci Rep 2018;8:10080. 10.1038/s41598-018-28162-x 29973671 PMC6031663

[46] Gaede L, Blumenstein J, Liebetrau C, Dörr O, Kim W-K, Nef H et al. Outcome after transvascular transcatheter aortic valve implantation in 2016. Eur Heart J 2018;39:667–675. 10.1093/eurheartj/ehx688 29228149 PMC5837346

[47] Sherwood MW, Xiang K, Matsouaka R, Li Z, Vemulapalli S, Vora AN et al. Incidence, temporal trends, and associated outcomes of vascular and bleeding complications in patients undergoing transfemoral Transcatheter aortic Valve replacement: insights from the Society of Thoracic Surgeons/American College of Cardiology Transcatheter Valve Therapies Registry. Circ Cardiovasc Interv 2020;13:e008227. 10.1161/circinterventions.119.008227 31937138

[48] Brønnum-Hansen H, Davidsen M, Thorvaldsen P. Long-term survival and causes of death after stroke. Stroke 2001;32:2131–2136. 10.1161/hs0901.094253 11546907

[49] Guertin JR, Feeny D, Tarride J-E. Age- and sex-specific Canadian utility norms, based on the 2013–2014 Canadian Community Health Survey. Can Med Assoc J 2018;190:E155–E161. 10.1503/cmaj.170317 29440335 PMC5809215

[50] Reynolds MR, Magnuson EA, Wang K, Lei Y, Vilain K, Walczak J et al. Cost-effectiveness of transcatheter aortic valve replacement compared with standard care among inoperable patients with severe aortic stenosis: results from the placement of aortic transcatheter valves (PARTNER) trial (Cohort B). Circulation 2012;125:1102–1109. 10.1161/circulationaha.111.054072 22308299

[51] Ontario Ministry of Health and Long Term Care . Schedule of benefits for physician services under the Health Insurance Act. Queen's Printer for Ontario. Ontario.; 2015; c2002. doi:

[52] Patient Cost Estimator . Canadian Institute for Health Information (CIHI). doi:

[53] Hospital Inpatient Care Case Costs—CMG/Plex—Open Government. doi:

[54] Mittmann N, Seung SJ, Hill MD, Phillips SJ, Hachinski V, Coté R et al. Impact of disability status on ischemic stroke costs in Canada in the first year. Can J Neurol Sci 2012;39:793–800. 10.1017/s0317167100015638 23041400

[55] Blackhouse G, Assasi N, Xie F, Gaebel K, Campbell K, Healey JS et al. Cost-effectiveness of catheter ablation for rhythm control of atrial fibrillation. Int J Vasc Med 2013;2013:262809. 10.1155/2013/262809 24089640 PMC3781920

